# Maintenance of certification for practicing physicians: a review of current challenges and considerations

**DOI:** 10.36834/cmej.53065

**Published:** 2020-03-16

**Authors:** Ligia Cordovani, Anne Wong, Sandra Monteiro

**Affiliations:** *1*Department of Health Research Methods, Evidence and Impact, McMaster University, Ontario, Canada; *2*Department of Anesthesia, McMaster University, Ontario, Canada; *3*Department of Health Research Methods, Evidence and Impact, McMaster University, Ontario, Canada

## Abstract

Maintenance of certification (MOC) has become increasingly important in medicine to ensure maintenance of competence throughout a physician’s career. This paper reviews current issues and challenges associated with MOC in medicine, including how to define medical competencies for practicing physicians, assessment, and how best to support physicians’ lifelong learning in a continuous and self-motivated way. We explore how the combination of self-monitoring, regular feedback, and peer support could improve self-assessment. Effective MOC programs are learner-driven, focused on every day practice, and incorporate educational principles. We discuss the importance of MOC to the physicians’ actual practice to improve acceptability. We review the benefits of tailored programs as well as decentralization of MOC programs to better characterize the physician’s practice. Lastly, we discuss the value of simulation-based medical education in MOC programs. Simulation-based education could be used to practice uncommon complications, life-threatening scenarios, non-technical skills improvement, and become proficient with new technology. As learners find simulation experiences educationally valuable, clinically relevant, and positive, simulation could be a way of increasing physicians’ participation in MOC programs.

## Abstract (fr)

Le maintien de la certification (MDC) revêt de plus en plus d’importance en médecine pour assurer le maintien des compétences tout au long de la carrière d’un médecin. Cet article examine les problèmes et les défis liés au MDC en médecine, notamment au chapitre de la définition descompétences médicales nécessaires aux médecins en pratique, de l’évaluation, et des meilleurs moyens d’appuyer la démarche d’apprenant à vie de façon continue et avec motivation intrinsèque. Nous explorons comment l’effet conjugué de l’autosurveillance, de la rétroaction régulière, et du soutien des pairs peut améliorer l’autoévaluation. Les programmes de MDC efficaces rendent l’apprenant actif, ciblés sur la pratique quotidienne, et ils intègrent également des principes d’enseignements. Afin d’améliorer l’acceptabilité, nous discutons de l’importance du MDC dans la pratique actuelle des médecins. Nous examinons les avantages liés aux programmes personnaliséset à la décentralisation des programmes MDC pour mieux caractériser la pratique du médecin. En dernier lieu, nous discutons de la valeur de l’enseignement par simulation dans les programmes de MDC. L’enseignement par simulation pourrait être utilisé pour pratiquer dans des contextes de complications rares ou de scénariosoù la vie du patient est menacée, à l’amélioration des compétences non techniques et à la maîtrise des nouvelles technologies. Avecles apprenants qui constatent que les expériences de simulations sont éducatives, cliniquement pertinentes et positives, celles-ci pourraient inciter les médecins à participer davantage aux programmes de MDC.

## Introduction

Several authors have written about the importance of continuing professional development (CPD), and maintenance of certification (MOC) in medicine in order to ensure maintenance of competence.^1^^–^^5^ Continuing professional development can be defined as multiple educational and developmental activities used to maintain or enhance professional skills and knowledge throughout a career.^6^^,^^7^ Competence, in the medical field, can be defined as the knowledge, skills, and attitudes that physicians should have to be able to take care of patients effectively.^8^^,^^9^ Some authors emphasize that the concept of competence is not static.^10^ Instead, physicians’ competenciesare dynamic, contextual, and developmental (over time). The terms “competence” and “competency” are synonymous.^11^ However, some authors describe “competence” as part of a person’s characteristic, and “competency” as an action of a person.^12^ We will use the term “competence” to mean both (a person’s characteristic and action).

Maintenance of certification programs refer to those programs that incorporate aspects of CPD to ensure the maintenance of competence throughout the physician’s career. Certifications have been used as quality indicators of hospitals, insurance companies, and physicians’ work.^1^ Although it is understood that maintenance of competence is needed, it is still not clear how best to design programs to ensure engagement from all physicians.^1^^,^^4^^,^^6^^,^^13^

After a period of training and experience, an individual’s skill performance becomes automatic and one is able to carry out a task without apparent effort.^14^ Dreyfus model of skill acquisition suggests that a learner progresses from novice to expert, advancing from rigid adherence to rules to intuitive understanding of situations.^15^ Automaticity may be a major advantage of experienced physicians. However, automaticity may impact conscious control over skill execution, making conscious movement correction challenging (particularly when one needs to modify a skill to incorporate new technology). Therefore, physicians might need to continuously work on avoiding arrested development from automaticity in order to improve performance.

Additionally, physicians need MOC actvitites to develop new skills as a consequence of advances in science and technology.^16^ Lastly, physicians’ practice often becomes narrow in scope over time, and MOC activities may be necessary to reinforce medical core competencies, to discuss areas where physicians have narrowed their practices, or to facilitate a change in area of interest.

In this narrative review, we explore the challenges of teaching practicing physicians within the context of the maintenance of professional skills and knowledge throughout a career. Specifically, we will discuss the challenges in defining medical competencies for practicing physicians, and how to best support physicians’ lifelong learning. Although some MOC programs include summative assessments,^17^^,^^18^ in this paper we will focus on exploring the formative learning activities of MOC. Therefore, we will not discuss remediation training (due to lapsed skills or behavioural issues) or clinical re-entry (when a physician is away from practice for a period of time).

### Literature search strategy

This article was, initially, a scholarly paper for the Master of Science in Health Science Education program at McMaster University (Canada). The topic was chosen due to the increasing awareness of the importance of CPD and MOC throughout the first author’s academic years in this program. The preliminary search of the literature included two databases (PUBMED and Web of Science) during the period of 1997 and 2017. First, we searched for the keywords *maintenance of certification, medical competencies, and continuing professional development*. The keywords *simulation and retraining, simulation and maintenance of certification* were added because we have soon noticed that several articles about MOC had included simulation in their discussions. Subsequently, specific citations from selected articles were retrieved. The relevance of each article (strengths, weaknesses, and limitations) was discussed among all the authors based on our expertise in the medical education field and medical practices. At the end of the process, we came to the agreement of discussing the challenges in defining medical competencies for practicing physicians, and how to best support physicians’ lifelong learning in a continuous and self-motivated way.

### Challenges in MOC

The challenges in effective MOC programing for practicing physicians include: defining the competencies, the assessment of competencies in practicing physicians, limitations of self-assessments, physicians’ engagement and motivation, and the relevance of MOC programs.

A main goal of MOC is to assure physicians’competencies throughout their careers; therefore, ensuring the quality of patient care.^13^ Medical competence frameworks, such as the CanMEDS (Canadian Medical Education Directions for Specialists) and the ACGME (Accreditation Council for Graduate Medical Education) Core Competencies, have been used in graduate and postgraduate medical education programs.^6^^,^^19^^,^^20^ More recently, medical competence frameworks have been incorporated into MOC programs focused on practicing physicians.^6^^,^^21^ For instance, the Royal College of Physicians and Surgeons of Canada has been developing the competence-based CPD program based on the CanMEDS framework.^22^^,^^23^ The idea is to use medical competencies to support lifelong learning. However, it might be difficult to define and measure competencies in practicing physicians.

As physicians’ practices become narrower in scope over time, the competencies defined by medical boards do not necessarily reflect the individual’s actual practice.^24^ As well, practicing physicians may lack a specific model in mind to compare their performances, and little work has been done to measure performances in physicians that have been working for a long period of time.^14^ For example, the Royal College intends to motivate physicians to pursue learning through activities arranged in three sections: group learning, self-learning, and assessment.^25^ There is also the MAINPORT ePortfolio,^26^ designed to be learner-driven and to help physicians to organize MOC activities based on one’s needs. The challenge faced here is the assumption that physicians are willing and able to identify their own training needs and seek out appropriate MOC activities. The limitations of self-assessment have been discussed in the literature.^27^^–^^29^ Therefore, ways to direct physicians to appropriate MOC activities should be identified.

Professional competencies defined by medical boards do not necessarily reflect the local needs of practicing physicians. Therefore, from a learning perspective, MOC programs based solely on CanMEDS roles might not engage physicians and might be perceived as a top-down approach instead of a learner-driven process.^24^ Strategies to overcome the challenge of improving physicians’ engagement in MOC programs could include tailored programs and activities that are relevant for their everyday practice.

In the following sections, we will explore strategies to overcome some of the previously cited challenges focusing on self-assessment (how physicians should choose what MOC activities to take in order to improve their practices), tailored programs (as a way to approach medical board’s requirements and local needs, as well increasing physicians’ willingness to participate in MOC activities), and simulation-based education (to promote physicians’ intrinsic motivation to learn).

### Considerations to overcome some challenges in MOC programs

***General*** ***educational*** ***principles:*** Deliberate practice (engaging purposefully in a well-defined task repeatedly) associated with feedback on daily medical practice has been found to be a key factor in maintenance and improvement of physicians’ performance.^7^^,^^13^^,^^14^ Other strategies to enhance learning are testing, distributed practice (practice interspersed with periods of rest) and mixed practice (frequent changes of task and examples).^30^^,^^31^ As well, reflective practice to mobilize relevant knowledge seems to be a key strategy for improving clinical reasoning.^30^ Lastly, research on memory demonstrates that physicians recall knowledge based on its deep meaning.^30^ Therefore, learning activities should create situations necessary for learning by emphasizing concepts and their deep meaning, and repeating the same information for several times in a short time frame. Maintenance of certification programs should incorporate the aforementioned strategies in order to enhance their educational effectiveness.

There are other practical suggestions in the literature for effective MOC programs. Learning activities should be based on needs of individuals, and should be learner-driven, and learner-centred.^1^ In other words, while physicians should take responsibility for their own learning, programs should be designed for performance improvement in a way that it is relevant, continuous and incorporated into their every day practice. Moreover, MOC activities should have clear goals with clear markers of progression towards these goals. The programs should be interactive and include sequenced tasks aiming at performance progression.^6^ Practice should be mixed, where different categories of examples or tasks are learned together.^30^^,^^32^ Additionally, blended courses (online and in-person) and simulation-based education could be included. Acquisition of technical skills should include well-established benchmarks to be achieved through deliberate practice and specific feedback. Preceptors could help to apply new knowledge and skills in the clinical settings.^33^^,^^34^ Preceptorship could be provided by a peer working at the learner’s institution, at the peer’s institution, or through short-period fellowships.

***Assessment and self-assessment:*** While physicians must be aware of their own limitations to decide their CPD plans, the limitations of self-assessment are well established in the literature.^27^^–^^29^ There are several possible reasons for this, including cognitive mechanisms that enable us to maintain a positive outlook on our abilities, and the fear of looking incompetent.^28^ Particularly important to physicians is that self-assessment might be limited due to the absence of a specific model of competence for comparison. In addition, self-assessment is not a fixed personal attribute, but varies depending on content, context, and perspective. Therefore, improving self-assessment include seeking feedback from multiple and varied external sources and taking the results seriously to improve performance.

Recently, research moved from the self-assessment of one’s overall performance to self-assessment during the performance (self-monitoring).^35^^,^^36^ These studies suggested that inaccuracy of self-assessment is partially due to the inability to evaluate performance over past events. Moment-by-moment self-monitoring seems to be more effective in indicating awareness of limitations in one’s competencies.^36^ Furthermore, reflection-in-action (self-assessment as a mechanism of ongoing monitoring, during the performance) seems to be better than reflection on action (after performance) for safe medical practice.^28^ Thus, physicians may be more capable of monitoring their skills in the moment of their performance, and then to determine their capability to solve specific problems.

One approach to lead physicians through MOC activities could be self-assessment based on external data, specifically aimed at the knowledge or skill in question.^27^^,^^37^ Physicians could engage with data through multisource feedback and reflection, following a plan to achieve their objectives and to measure outcomes after change. In fact, multisource feedback has been adopted as part of the Royal College of Physicians and Surgeons of Canada’s CPD activities.^38^ However, it was suggested that regular smaller ongoing assessments, instead of a single opportunity, would better help physicians to identify areas for improvement.^39^ Moreover, peer assessment in real-time may be a helpful approach.^7^^,^^42^^–^^45^ A physician might feel better supported by a colleague who has a similar practice. The alignment of self-monitoring, regular feedback, and peer support could be an approach that helps physicians to overcome the challenges of self-assessment, discloses possible skills gap, and leads physiciansthrough relevant MOC activities.

***Tailored programs:*** Professional competencies defined by medical boards and organizations do not necessarily reflect the individual’s actual practice.^24^ For example, an anesthesiologist working in a small community clinic would not practice as an anesthesiologist working in a large cardiac centre. Moreover, it seems that MOC activities would be more acceptable to physicians if they perceive the program’s relevance to their everyday tasks. Therefore, tailored programs seem to better characterize the physicians’ practices, thus, increasing acceptability.^24^^,^^46^^,^^47^

New technologies could help to develop a more individualized program, increasing validity and reliability, and decreasing costs at the same time. For instance, the University of Ottawa Anesthesia Residency Program developed an online resident-driven assessment tool called Clinical Case Assessment Tool (CCAT).^48^ The residents enter identified data about their real cases. Then, the resident reflects about one’s performance. Next, the data are shared with the faculty for face-to-face feedback with the resident. Physicians could use online tools, such as the CCAT, to record data from their cases that could be easily analysed. Physicians could engage with the data through reflections and feedback from a peer. Electronic format (from charts, audits, regional or national databases) allows easy data analysis for assessment and improvements as well as customized programs.

The main concern regarding tailored programs is that medical competencies that are considered essential regardless of actual practice characteristics may not be included. Therefore stakeholders’ expectations (medical boards, hospitals, patients) of physicians’ competencies might not be evaluated.^24^

Perhaps the solution for this gap between the actual practice and the societal expectations might be a regional program based on local needs. Local institutions (cities, universities, hospitals) could develop MOC activities based on boards’ regulations but reflecting the community expectations as well as the physicians’ everyday practice. Local surveys addressed to a specific population might help to reflect the community needs.^49^^,^^50^This could not only contribute to increased public satisfaction with the health system, but could also contribute to increase physicians’ participation in MOC activities once they perceive intrinsic benefits to their patients.^41^

Clinical doubts generated by physicians (self-reported or directed observed) during the care of patients could provide meaningful topics for MOC activities.^51^^,^^52^ These doubts might be a consequence of the moment-by-moment self-monitoring and reflection in action discussed before. The questions generated during the physicians’ performances are more likely to be practice-relevant and practice-changing. The collection of questions and topics could be contrasted with local patients’ needs and national boards’ expectations (e.g. the CanMEDS).

Therefore, a possible way to include tailored programs would be through the decentralization of MOC programs (for instance, partnering with local entities on adapting MOC programs). The activities should target at a specific population of physicians and community but using national boards’ directives as an overall framework.

***Simulation-based education in MOC:*** Simulation-based medical education has become widely accepted in medical residency programs and MOC programs around the world.^19^^,^^53^^–^^57^ In the US, simulation-based education is encouraged for anesthesiologists looking for recertification.^54^^,^^56^^,^^58^ In Canada, the Canadian National Anesthesiology Simulation Curriculum (CanNASC) has been designing scenarios to reflect clinical situations that are critical to the competence of an anesthesiologist.^19^^,^^59^ Still, simulation-based education to teach practicing physicians could receive more attention.

Despite some concerns about the quality of the literature supporting simulation-based education,^60^^–^^62^ the use of this approach for MOC is valuable for a few reasons. Simulation can explore learning needs that are relevant to trainees’ experiences.^63^ Simulation is able to promote feedback to foster reflection, discussions of concepts, and repeated practice. Moreover, simulation programs can be customized. A tailored program, as previously mentioned, can increase acceptability by physicians and enhance learning.^64^ Additionally, simulation can be offered in different modalities (e.g. standardized patient, mannequin, part-task training, and theatre-based simulation). Therefore, different instructional methods can be used depending on the learner’s needs.^65^^,^^66^

Simulation is ideal for deliberate practice, and practice can be sequential, distributed and mixed. Simulation is a safe learning environment. Confidentiality, trust, and credibility are paramount. Errors are expected and permitted. Therefore, simulation could help physiciansto overcome the fear of being judged, especially when a psychologically safe learning environment is created.^67^ Effective learning approaches, such as reflection-in-practice and immediate feedback, are easier done in simulated scenarios than in real life, especially in uncommon complications and life-threatening situations.

Clinical skill acquisition is a common learning objective of simulation.^65^ Simulation could be used specifically to practice rare cases involving the need to make quick decisions in dynamic settings.^68^^,^^69^ Simulation has also demonstrated utility as a tool to teach non-technical skills, such as teamwork.^61^^,^^65^^,^^70^^,^^71^ The harm-free learning environment gives opportunity to practice common and uncommon scenarios. Another specific use for simulation is to teach new procedures related to new technologies or changing scope of practice (e.g. ultrasound, and video laryngoscopy).

Probably the greatest (and less commonly explored) advantage of including simulation in MOC programs is that the learners usually find the simulation experiences to be educationally valuable, clinically relevant, and positive.^55^^,^^60^^,^^72^ Although some authors discuss the negative effects of participants’ anxiety that may precede a simulation experience,^60^^,^^73^^–^^75^ it seems that the level of stress is associated with prior experience with simulators (less experience contributes to higher stress). It is expected that participants’ anxiety will be alleviated with time since educational programs are increasingly incorporating simulation and physicians are becoming more used to this approach. Motivational factors appear to be the key to increase MOC participation.^6^^,^^41^^,^^67^^,^^76^ The positive simulation learning experience could help physicians’ motivation to participate in MOC activities. As discussed elsewhere,^6^ MOC programs should focus on the joy of learning instead of being driven by external regulations. Simulation in MOC programs could include medical boards’ directives, and still be a positive experience for the learner.

## Discussion and recommendations

In the first part of this paper we explored some challenges of MOC in medicine (see Appendix for key points). With the increasing emphasis on competence-based medical education, MOC programs have been changing the focus toward achievement of competencies. Therefore, the first significant challenge might be to define expected medical competencies in practicing physicians with a specific scope of practice. This is compounded by a lack of a standardised model of competence for comparison. Another important challenge is motivating and supporting physicians in searching for effective MOC activities. We discussed strategies to detect the existence of a gap in physicians’ performances as a way to overcome those challenges. We suggest that the convergence of self-monitoring, regular feedback, and ongoing peer support could be an educationally effective approach that would be acceptable by physicians and medical boards. Once the gap is identified, other strategies should focus on how to offer MOC activities in order to promote guidance and motivation to learn throughout the professional life.In this regard, we suggest the decentralization of MOC programs to particular contexts but including national boards’ directives. Activities should aim at a specific population of physicians and community, based on patients’ expectations of physicians and on physicians’ generated topics.

Lastly, we discussed the use of simulation-based education as part of MOC programs. We suggest emphasizing the positive simulation experience described by physicians as a way to increase participation in MOC activities. We recommend that simulation should be used to practice rare complications and life-threatening situation scenarios, teamwork skills improvement, and to introduce new technical skills.

Barriers to carrying out these suggestions are to be expected. First, any educational program implementation is a challenge and effective leadership is one of the most important factors for successful achievement. Second, specific programs limit standardization and generalizability. Thus, each institution should balance time, cost, and benefits of offering local MOC activities. Third, although ongoing peer support seems to be an effective approach, the best way to pair physician-peer is unknown. More investigations regarding this topic is needed. Lastly, simulation-based education can be costly. Considerations regarding the type of simulation to be used could help with this issue. Furthermore, considering the current attention to competence-based education, the benefits and challenges of using this approach in MOC programs should be a topic for further research.

### Conclusion

Competence-based MOC programs are critical for practicing physicians. Although the idea of lifelong learning needs to be accepted by all physicians, the best way to do that in order to achieve and enhance medical competencies is not clear. It does not seem reasonable to place the onus and the responsibility only on physicians. Instead, MOC programs should concentrate efforts on incorporating educational principles, improving self-assessment strategies, and defining competencies that meet the profession’s standards while including meaningful topics that are relevant tophysicians’ everyday practices and communities.

## Appendix A.

### Key Points

**Review of the challenges in Maintenance of Certification for practicing physicians**

- Defining the competencies in practicing physicians
- Assessing competencies in practicing physicians
- Limitations of self-assessments
- Physicians’ engagement and motivation
- The relevance of MOC programs for practicing physicians

**Recommendations to overcome some of the challenges**

- Improving physicians’ assessment and self-assessment to help physicians to choose relevant MOC programs
  - Feedback from multiple and varied external sources
  - Moment-by-moment self-monitoring
  - Reflection-in-action
  - Small ongoing peer assessment
- Increasing physicians’ engagement and motivation
  - Tailored programs
  - Decentralization of MOC programs
  - Meaningful topics for MOC activities through clinical doubts generated by physicians
  - Simulation-based education in MOC activities
